# Aging as Otherness: Revisiting Simone de Beauvoir’s *Old Age*

**DOI:** 10.1093/geront/gnab034

**Published:** 2021-03-04

**Authors:** Chris Gilleard

**Affiliations:** Division of Psychiatry, Faculty of Brain Sciences, University College London, London, UK

**Keywords:** de Beauvoir, Heidegger, Later life, Otherness, Sartre, Unrealizability

## Abstract

Aging has been given short shrift as a topic in philosophy. The aim of this article is to redress this neglect by revisiting some of the key philosophical issues in Simone de Beauvoir’s book, *Old Age*. In her notion of old age’s unrealizability, its impossibility of fully embodying a subject position, and the role played by the other in denying such subjectivity, she draws upon the work of both Heidegger and Sartre. The dilemma she repeatedly draws attention to, of always seeming to age in ways other than as one’s self, raises the question of whether any view of aging as an authentic subjectivity may be no more than, in Heidegger’s words, a “chimerical undertaking.” In examining how the concepts of bad faith and inauthenticity are used by Heidegger and Sartre, the article concludes that for both these writers, an authentic subject position can be maintained in later life, without ending up as the otherwise inauthentic subject of others’ collective imaginary of “a good age.”

While philosophers think a lot about death, they think much less about aging and old age (Bavidge, 2016, p. 207; Mothersill, 1999, p. 9). As Helen Small has put it, philosophy has shown itself “far more interested in ‘mortal questions’” than in the business of growing old (Small, 2007, p. 1). A notable exception is the French writer, Simone de Beauvoir, whose book, *Old Age*, stands out almost alone in exploring aging as a distinctly philosophical issue (de Beauvoir, 1977). Although the broadcaster, essayist, and philosopher, Jean Améry, wrote a book about aging about the same time, echoing, or rather anticipating, many of the themes developed by de Beauvoir, including the inherent otherness of age (Améry, 1968, 1994), his book was not translated into English until long after the author’s death. What unifies both writers is that they saw little to appreciate in old age, viewing aging as a process of increasing alienation from one’s self and from the wider society: in short as generalized othering.

While both Améry and de Beauvoir also recognized that older people were given little value by the wider society, Améry chose merely to list and illustrate the forms and manner that such alienation took and the way it was realized within society. de Beauvoir sought to go further, to understand what it was about aging that led to the “double” alienation that she felt aging encapsulated, of becoming both other to oneself and being othered by society. In formulating her position, she drew upon the existentialist phenomenology of Heidegger and Sartre, and the importance of the “Look” that with age would gradually overshadow the voice of intent and self-sameness (Dolezal, 2012; Gothlin, 2006). For de Beauvoir, this meant that sustaining an “authentic” subjectivity as one grew old was effectively ruled out as “unrealizable.” Whether such a conclusion is warranted from this philosophical position requires going outside de Beauvoir’s book to reconsider the framework within which she drew her position, namely, the writings of Sartre and Heidegger. This is the central aim of this article, not just to revisit de Beauvoir’s book, which has been done before (Woodward, 2016) but to reexamine its central thesis concerning the inherent otherness of age in the light of these other texts. Before exploring these possibilities, the article first outlines the philosophical concepts of “otherness,” “selfhood,” and “unrealizability,” as they are employed by de Beauvoir in her book, *Old Age*. Although some of these concepts were probably developed mutually by de Beauvoir and Sartre, the first section focuses upon her own account, before turning in the second section to the broader existentialist/phenomenological positions regarding self and other as set out in Sartre’s book, *Being and Nothingness*, whose thesis de Beauvoir made central in her account of “the discovery and assumption of old age” (de Beauvoir, 1977, pp. 315–402). Then in the third section, I reconsider in more analytical detail de Beauvoir’s approach to the problem of the “unrealizability” of age, its relationship to the “other within the self,” and the links it bears with Sartre’s discussion of self and others. In the fourth section, I reconsider de Beauvoir’s view of aging and agedness as ontologically unrealizable subject positions, and what this might mean for the study of age and aging and the (im)possibility of age acquiring (realizing) its own “authenticity.”

## Aging as the Unrealizable Other: de Beauvoir’s Core Thesis

While the first part of *Old Age* concerns old age as a social phenomenon ignored and marginalized within society, the philosophical core of her book begins at the start of the second half of her book, where her notion of the intrinsic otherness and unrealizability of age is described. There is, she argues, “an insoluble contradiction between the obvious clarity of the inward feeling that guarantees our unchanging quality and the objective certainty of our transformation” (de Beauvoir, 1977, p. 323). While there exist “an infinite number of ways” of taking upon one’s self the external reality of “old,” of aging, “not one of them will allow myself to coincide with the reality that I assume. Old age is … something of which I cannot have any full inward experience” (de Beauvoir, 1977, p. 324). It is subjectively an “unrealizable,” realized only in and through myself in others’ eyes.

Heinämaa suggests that the tension de Beauvoir here is referring to arises from “two different forms of self-relating, one proceeding by immediate experience and the other constituted via relations with other subjects” (Heinämaa, 2014, p. 171). This corresponds with Sartre’s analysis of selfhood, in *Being and Nothingness*, between my “being-for-oneself” and my “being-for-others” which corresponds in turn with “my being looked at” (Sartre, 2003, p. 300). As Heinämaa observes, while this tension can be understood as a necessary part of what she calls “the ambiguity of our existence,” the process of aging and the external transformation it brings about bring this ambiguity acutely to the fore (Heinämaa, 2014, p. 172). With age, an unbridgeable gap is revealed between these two forms of selfhood, between what de Beauvoir terms “the other-in-me” or the me that exists in and through the other, that is through “the Look,” and the “for ever present me,” the central unchanging focus, the lens that is my experiencing. Even as the look of one’s age arises first through my being observed by others, de Beauvoir recognizes that “my being looked at” also forms part of my being in the world, as the other within me, without which there can be no “me” to experience the world. The divide, in short, is not one between the out there—society—and the in here—self—but resides, differently but just as surely, in here, between my being-for-myself and my being-with-others.

## Conceiving “Self” and “Otherness”: Sartre’s *Being and Nothingness*

A more detailed analysis of this divide between the experienced and the experiencing “me” is to be found in Sartre’s book, *Being and Nothingness*, written while he was a “prisoner of war” after the German invasion of France and published in occupied Paris (Sartre, 2003). To better frame the concept of “otherness” as it is used by de Beauvoir and as it is employed in this article, I will briefly outline the main arguments Sartre puts forward in the third section of this book, entitled *being-for-others* (Sartre, 2003, pp. 243–452). Here Sartre discusses the existence of “the other” and the critical role the other plays, first in distinguishing between what Sartre called the self in its “for-itself” mode and the self as it exists in its “in-itself” mode (Sartre, 2003, p. 246). For Sartre, these two aspects of self—its “for-itself” and “in-itself” modes—form the twin axes around which existence is realized. The self in its “for-itself” mode is the central focus animating consciousness, while the self “in-itself” is the sense of our being a person in the world, through the multiple materialities forming our embodied being. While the latter is a presence in the world, the “for-itself” is defined, at least in part, by its “perpetually determining itself not to be” its “in-itself” ness (Sartre, 2003, p. 109). When another person observes us, they can only observe our “in-itself” ness, of course, not our unwavering “for-itself” ness, but by being acknowledged as another, our “in-itself” ness becomes both the object of another’s consciousness, and another subject endowed with the appearance of a fellow sameness. Sartre’s point is that left to our consciousness alone, our “in-itself” existence would not, in effect, be alienated from our inner sense of being. The gap between our inner and our outer self arises only by our awareness of being the object of another’s consciousness, of being a consciousness that does not fully own itself, that is realized, in part, in and through others’ eyes, what Sartre calls “the Look” (Sartre, 2003, p. 293). That our “in-itself” ness can be recognized as “other” opens up the possibility of alienation, of being other than one’s self. This other pierces our consciousness “to the deepest part of its being” making our “being-for-others” “a necessary condition” for our being-for ourselves (Sartre, 2003, p. 262). This is what de Beauvoir means by “the other within us” (de Beauvoir, 1977, p. 321).

Sartre rejects what he sees as Heidegger’s formulation, that our being “for itself” can only arise through our being “with others”; that existence is effectively formed through shared existence and existence, through shared experience. Instead, he argues that it is the capacity of the other to be experienced as looking, in the same way as we look, that renders the other incapable of being experienced as mere object-ness (Sartre, 2003, p. 288). The importance of the other’s look lies in the fact that through it, “I am looked-at in a world that is looked-at,” experiencing myself in my “object-ness,” an “unrevealed object-ness” that in turn reveals “the inapprehensible subjectivity of the other” (Sartre, 2003, p. 294). Although we cannot experience the interiority of the other in the way that we can experience our own “for-itself” ness, we can still sense, in our being, a common subjectivity that renders the other more powerful in shaping our own being than were there to be no such commonality, merely a “nonsubject” like object, lacking the power of othering that is conferred by the “Look.” Being with others and being looked at by others both constitute and constrain us.

Sartre then turns to the role played by the body in realizing both the other’s object-ness and our own. He begins by distinguishing two aspects of the body—its “in-itself” ness, its being an object in the world to which others have at least as much access, if not more, than the self does, and its existence as a necessary part of my being, the being of my body for me, the material constitution of my personhood. The body as a “being-for-others,” he states, is of a different order to the body as a “being for itself” (Sartre, 2003, p. 329). The former can only ever be an object of consciousness, while the latter is a necessary part of my subjectivity, of how I exist. The incomparability between these two registers of “body-ness” leads Sartre to conceive of consciousness of our body functioning in two different ways, one as it serves as a conscious object for others (he gives the example of watching a physician examining him and stating how he sees the physician’s body and the body the physician is examining as alike, objects in themselves) and the other as the consciousness of a sign, something that though it can be observed as a particular shape and form, is more usually ignored as no more than a “way station” for the meanings, ideas, and imaginings that have little to do with the body’s material form. The body-for-itself, he argues, is like a sign providing an affordance to some action, its physical qualities being immaterial to the self’s intent. We read a page; our eyes can be observed moving back and forth, dropping a little lower as our reading progresses down the page. The body in its “for itself” ness is engaged with the text—the argument, story, or thesis put forward on the page—which is of a different nature to either the marks on the page or the movements of the eyes (Sartre, 2003, p. 355). The two bodies, the reader’s and the reading, coexist but are incapable of coalescing as a conscious unity; they perform two different registers. A “third ontological dimension” of the body also exists, beyond my existing as my body and as a body known by others, whereby “I exist for myself as known by the other,” in a “depth of being which is for me [a] perpetual ‘outside’ of my most intimate ‘inside’” (Sartre, 2003, p. 375). This third dimension exists because of the omnipresence of the other; our embodiment realized through the other, revealing, as he put it, “the emptiness of the existence of my body outside as an in-itself for the other” (Sartre, 2003, p. 375). Neither the existing conscious body nor the body observable within a world of objects, this third dimension exists solely through and in the eye of the beholder, “a point of view on which are brought to bear points of view which I could never take” (Sartre, 2003, p. 375). It is at the same time a consciousness of the body, a corporeal self-consciousness that always and only exists as it appears to the other, a body locked within the look. This is where de Beauvoir places age, neither a part of our embodied “for-itself” ness nor merely the objective character of our body’s aged “in-itself” ness, still recognizably my body but alienated, from the start from a fully realizable “for itself” ownership, and which old age only alienates further.

## Revisiting Age’s “Being in the World”

de Beauvoir’s *Old Age* treats old age as just such an “in-itself-for-others,” and our aged body as “the body-for-us, but inapprehensible and alienated” to use Sartre’s words (Sartre, 2003, p. 377). Old age’s otherness raises obstacles to the body as part of the signifying conditions through which we realize our plans and projects. Rather than through our body realizing its “for-itself”-ness, age, old age appears as an externality, as “something that just happens” (de Beauvoir, 1977, p. 313). The absence of intent, of self-directedness, is for her crucial in denying old age any subjectivity—any capacity to exist and be realized as a “for-itself.” The unrealizability of any “for-itself” old age reflects old age’s inherent otherness, its emergence always and only in its “in-itself-ness,” something come upon, realized through the gaze of the other, and appearing within that third ontological dimension that Sartre delineated in his examination of the body and its role in human being—as a body-for-others.

de Beauvoir does not leave it there. She recognizes that the body—in its “in-itself-ness”—changes, acquires the “look” of age, not through any intention or agency but through a look that is realized in and through the other (less a specific other than the generality we have of others’ consciousness). These looks—these confrontations with the other who, though object-like, is recognized as another consciousness, another subject, sharing in the point of view of the self-for-others that constitutes our intersubjective reflected self—fuel, as de Beauvoir puts it, the other accumulated within. For de Beauvoir, it is this accumulation of looks by which our old age is realized, not through any for-itself realization but the result of a succession of looks which despite their original otherness are gradually acceded to, owned, to a degree, as my body-for-others, but never realized, never owned as fully mine, as my existing.

Before the onset of old age, de Beauvoir says, the person we are to the outside world “is as many sided as the rest of the world itself” and no one viewpoint of ourself “for others” prevails. Our “self-as-other” can be challenged, contested, one facet turned to, as another facet is turned against (de Beauvoir, 1977, p. 316). With the onset of age, there are fewer facets to turn to, and more to turn against, as they bear the multiple signs of aging. While denial or rejection, struggles, and refusals continue, our being old for others gradually overwhelms the possibility of our being-old-for-ourselves; and as our body becomes more a body for others than for ourselves, so our subjectivity—our being-for-ourselves—is slowly subsumed beneath our embodiment for others.

While de Beauvoir continues to employ the distinction between an embodied consciousness that exists as a for-itself body and an embodied consciousness that exists “in itself” as an object consciously recognized as existing independently, outside of consciousness, her focus is very much on the struggle within, that she sees occurring between the subjective and objective poles of conscious aging. In the many accounts, anecdotes, and autobiographical sketches that she draws upon in charting how aging and old age are talked about as first-person experiences, her constant theme is the conflict to either own or disown one’s old age identity. The subject accounts she describes reflect this internal struggle between the need and the inability to fully realize “aging”; not simply to “own” it, but to really be it, to be really old and in aging to continue to become my existing for-itself body. The nearest she seems to get is what she calls the “assumption” of age, which, to this reader, at least, seems to mean something like an acceptance of (or submission to) the other within; like acknowledging resignedly that one has become the body that one’s body is for others. If not fully a “for-itself” body, the aging body becomes a compromise, between our being and our becoming, a step-self perhaps, but bending to what has been called age’s “arc of acquiescence” (Higgs & Jones, 2009, p. 85).

## Subjectivity and Authenticity in Aging

If de Beauvoir is right, and the only form of subjective aging is that achieved by renunciating the body-for-itself and acceding to the subjectification represented by Sartre’s third ontological dimension, of becoming a body-for-others, does that mean that with aging, the body’s “most intimate inside” must become a perpetual outside (Sartre, 2003, p. 375)? The dilemma de Beauvoir poses in her book is that because old age is unrealizable in any “for-itself” mode, it can only ever be realized as aging through others—as a body-aging-for-others that over time we learn to accede to, but never fully realize, as our own. Does this mean that we always must age with concessions to the otherness within and without that age brings—that we must always age by acceding to the other within, that is with a degree of inauthenticity, of bad faith?

It is difficult to believe that de Beauvoir herself saw any resolution to this problem. But is her conclusion concerning the impossibility of living authentically in later life an inevitable consequence of accepting de Beauvoir’s and Sartre’s existentialist philosophy? In this final section, I want to turn to this concept of authenticity, as it was laid out by Heidegger and in the existentialist phenomenology that she and Sartre developed from reading Heidegger’s work, to interrogate de Beauvoir’s position. Although Sartre focused more upon bad faith—inauthenticity—in his writing than he did upon authenticity, concepts of “authenticity,” “bad faith,” and “ownedness” are all linked terms whose point of origin lies with Heidegger’s writing (Gothlin, 2006).

Heidegger’s *Being and Time* introduced the concept of authenticity into phenomenology (Heidegger, 2010, p. 53). For Heidegger, our existence can only ever be an existence in the world; it cannot be abstracted from this (e.g., as a “pure” being) nor can it be understood without reference to the existence of others. There is no pure subject without such existence, and no existence in the world without it shares the world of others’ existence. Human reality, Heidegger’s famous *Dasein*, only becomes “authentic” to the degree to which we experience “care” in the sense of our being concerned with our existence, of our being invested in being in the world, not through imagining we can escape it (Heidegger, 2010, p. 185). Our being in the world, including this concern for our being, of caring to be, does not of course guarantee authenticity; *Dasein* is always “free for authenticity or inauthenticity” (Heidegger, 2010, p. 223). To realize authenticity, then, is to seek to realize the completeness of being, a task which Heidegger acknowledges as being always and only ever a potentiality, perhaps, he says at one point, even “a chimerical undertaking” (Heidegger, 2010, p. 249).

This realization amounts, in one commentator’s mind at least, “to ‘owning up’ to that essential nullity in an attitude of openness and resolve” (Carman, 2000, p. 13). This resolve means rejecting—or overcoming—what Heidegger calls a “they-self” way of being (Heidegger, 2010, p. 257) for a realizable ownership of one’s own being, without any illusions of its possessing an “internal” essence or intrinsic direction awaiting our discovery. To achieve such a potentially authentic existence requires finding a way of being one’s own self—choosing to make our choices as a being that is “ontologically different in kind from things present in the surrounding world” (Heidegger, 2010, p. 259). The risk of not choosing is falling into alienation through an entangled being in the world, “plunging into the groundlessness and nothingness of inauthentic everydayness” (Heidegger, 2010, pp. 171–172).

Sartre reframed Heidegger’s authenticity through his concept of “bad faith.” For Sartre, “to constitute ourselves as being what we are … by that very positing we surpass this being—and not toward another being but toward emptiness” (Sartre, 2003, p. 86). While we can constitute ourselves historically, he argues, by what or whom we have been, all attempts to constitute ourselves as we are constituted merely the grounds of bad faith (Sartre, 2003, p. 92). The past, Sartre argues, “is without force to constitute the present and to sketch out the future” but at the same time “the freedom which escapes toward the future cannot give itself any past it likes” (Sartre, 2003, p. 517). The fixity of the past is precisely that; it does not define our being in the world, nor does it determine our becoming. Denying the forever present open-ness of existence is, however, hard to do, let alone maintain, for even the most resolute of persons. A pervasive presence of “bad faith” is, for Sartre, almost an inevitable consequence because consciousness is always fated “to be what it is not and not to be what it is,” a game of mirrors, an intentionality always in flight (Sartre, 2003, p. 94). Being anyone—young or old, truthful or dishonest, man or woman—on the principal grounds of having been someone risks bringing down bad faith because it denies the ever-presence of agency, of our always becoming, always on the move toward another becoming.

de Beauvoir sees aging as limiting the opportunities for becoming. For her, aging rather risks sinking, contentedly or otherwise, into a position of bad faith, giving up on becoming and resting instead upon what one was and what, in others’ eyes, one now is. For her, the unrealizability of old age—the inability fully to own it, fully to be it—constitutes the grounds for aging in an unavoidable bad faith, through the force of past circumstance and the increasing presence of the other within us. In a recent article on the topic of authenticity and aging, Hanne Laceulle has argued that what she terms “authenticity discourse” is “capable of acknowledging the positive potentials of growth and development that later life may harbor” (Laceulle, 2018, p. 970). Such an optimistic reading of authenticity certainly reflects the interpretation of Heidegger that Guignon has made—that authenticity involves facing up to the inevitable truth of one’s own finitude and living each moment “as an integral component of the overall story it is shaping in its actions” (Guignon, 2000, p. 89). This narrative approach to authenticity, however, is for some problematic, because it relies upon language and language is part of the already existing world, a product of *das Man* (the Anyone), such that all narratives, all stories risk recapitulating the “reifying and banalizing forces inherent in discursive practice” (Carman, 2000, p. 24).

If authenticity involves taking responsibility for one’s self, balancing the weight of the past with the weightlessness of the future to forge an authentic present, such resolute open-ness was, in de Beauvoir’s eyes, a task that with age becomes less possible. With age, we become hemmed in, both by the body’s in-itself-ness and by its representation both as the other within us and the other without, which together conspire to overwhelm the desires, the intentions, and projects of our continuing, resolutely, to become. Becoming old, in short, is a way of framing the past that is shaped by and shapes our feeling ourselves old and which in turn shapes our imagined future. We risk making of ourselves a third-person narrative, a “me-story” that is little more than a “they-story,” denying or refusing to acknowledge the openness that is the present. Viewing one’s old age as a “completed life,” a story told, is perhaps less a mark of narrative authenticity than of bad faith, a lack or loss of what Heidegger called “resoluteness.” Denying one’s having become old and denying one’s intentions to continue becoming older may equally be examples of bad faith, but, within Sartre’s existential phenomenology, there is no necessity that they should be so. This is because, for Sartre, “the for-itself cannot be anything.” All representations of what I am can only be applied to my past and my person. They cannot be what I am nor determine what I intend becoming. Accepting our entanglement within a past and a future, but abdicating the freedom entailed in such “being-for-itself” seems archetypally bad faith, inauthenticity.

Capitulating to the aging other instead of grasping such characteristics as age “only in the light of my own ends” and giving them “a meaning which my freedom confers on them” is, on the other hand, a demonstration of authenticity, of positive faith. As Sartre says, these unrealizables “are for the other but they can be for me, only if I choose them … [and] so be for myself … by choosing myself such as I appear to the other” (Sartre, 2003, p. 550). Applied to what being old authentically might mean, Sartre seems to suggest that it is being free to be old in a way of one’s own choosing, of realizing oneself becoming older in the light of, but not as subject to, the other. This perhaps is the most one can strive for in achieving “one’s own factical particularity” (Carman, 2000, p. 21).

## Conclusions

The aim of this article has been first to outline de Beauvoir’s central proposition concerning age’s unrealizability and second to interrogate her thesis through the writings of other existential phenomenologists who were key in developing her position. As noted at the outset, within philosophy, inquiries into old age and aging have been, and remain, quite limited, at least outside the realm of ethics. Increasingly, however, Simone de Beauvoir’s book on old age has come to be recognized as a key text challenging such generalizations (Stoller, 2014). Within aging studies and gerontology, despite the humanities and arts expanding their intellectual and institutional presence (Achenbaum, 2020, p. 594), such advances have come about primarily through the arts, literature, and media studies—and to some extent historical studies. The presence of philosophy in aging studies is almost as uncommon as its inverse, the presence of age in philosophy. Again, de Beauvoir is the notable exception.

In choosing to revisit (again) de Beauvoir, my aim has been not simply to undertake another re-reading of her book and what it tells us about her and her times. Rather, it has been to focus, more narrowly, upon some of the core philosophical issues she raises concerning the experience of aging. Her central thesis was that the process of aging is one of “othering,” both the othering that takes place without—in society and its institutions—and the othering that takes place within. For de Beauvoir, aging thus constitutes a form of double alienation that leads to an unbridgeable gap between what de Beauvoir and Sartre called the self as a “for itself” and the self as an “in itself” and as a “for others.” This gap—between what might be called the “subject pole” and the “object pole” of experience—de Beauvoir considered grows inexorably with age, leaving the only option to draw down the arc of acquiescence; to accede to this othering from within, though clearly also resisting any acquiescence to the othering from without.

While it is possible to reframe these ideas under the rubric employed in studies of “objective” and “subjective” age (Montpare, 2009), this would be, in my view, a mistake. For within this paradigm of subjective and objective age, age remains a characteristic more attributed than realized, whose attributions are “entangled” in us and in the world. In themselves, such studies provide little evidence to accept or reject de Beauvoir’s thesis of age’s unrealizability. On the other hand, one can interrogate her thesis along somewhat different lines, through the concept of authenticity (and bad faith) as outlined in Heidegger’s and Sartre’s writings. While Heidegger criticizes the inauthenticity of ways of being that deny one’s being in the world, he likewise assumes that ways of being in the world realized as and through others may prove equally inauthentic. Such ways typically reflect choosing an “anyone” mode of being rather than owning one’s “factical” being, as necessarily open and necessarily particular. For Heidegger, the task (whether or not it may prove ultimately chimerical) is to turn toward the possibility of becoming, and not let human reality “surrender [-] itself to beings which it itself is not” (Heidegger, 2010, p. 171).

Sartre’s account of the possibilities of being, of being both free to become and at the same time owning what one has already become, suggests a similar alternative, implying that aging—becoming old—is capable of sustaining authentic “for-itself” intentions. In his discussion of the freedom encompassed by and realized within our subjectivity—our being-for-ourselves—Sartre suggests that we can own the characteristics that otherwise entangle us. Owning and giving meaning to those “unrealizables,” choosing the meaning we give to our being—whether being old or any other characteristic attributed to people by people—represents an option not for conferring authenticity upon age as some abstract ethical category applied to “they-selves,” but through our own choosing a particular way of living a “for itself” later life. In sum, neither to deny nor embrace aging, but fashion it to our ends. The humanities, as de Beauvoir well recognized, and literature, in particular, may offer examples both of how such later lives may be realized and equally how old age can be subsumed by inauthenticity, through acceding to the other within, as much as by submitting to the othering of society and its institutions.
